# Postoperative Pain Outcomes and Satisfaction in Preschool Versus School-Age Children: A Prospective Multicenter Observational Study

**DOI:** 10.7759/cureus.109502

**Published:** 2026-05-23

**Authors:** Salah N El-Tallawy, Joseph V Pergolizzi, Abdullah T Alsubaie, Mohamed M Hegab, Aziz U Haq, Ahmed M Abd El-Rahman, Mohammed L Helmy, Rizwan A Khan, Hatem H Maghrabi, Amjad R Rasheed, Dalia A Helal, Al-Shabrawy M Tawfik, Samah S Elzakaziki, Mohamed M Alharbi, Aya A Mohyeldin, Bilal M Delvi

**Affiliations:** 1 Anesthesia and Pain Management, Faculty of Medicine, Minia University and National Cancer Institute, Cairo University, Cairo, EGY; 2 Anesthesia and Pain Management, King Khalid University Hospital College of Medicine, King Saud University, Riyadh, SAU; 3 Anesthesia and Pain Management, NEMA Research Inc, Naples, USA; 4 Anesthesia and Pain Management, King Abdulaziz University Hospital, King Saud University Medical City, Riyadh, SAU; 5 Anesthesia, Critical Care, and Pain Management, Al-Azhar University, Cairo, EGY; 6 Anesthesia, Dental University Hospital, King Saud University Medical City, Riyadh, SAU; 7 Anesthesia, King Abdulaziz University Hospital, King Saud University Medical City, Riyadh, SAU; 8 Anesthesia, Critical Care, and Pain Management, King Abdulaziz University Hospital, King Saud University Medical City, Riyadh, SAU; 9 Nursing, King Abdulaziz University Hospital, King Saud University Medical City, Riyadh, SAU; 10 College of Medicine, Kasr Al-Aini Hospital, Cairo University, Giza, EGY; 11 Anesthesia and Pain Management, King Khalid University Hospital, King Saud University Medical City, Riyadh, SAU

**Keywords:** multimodal analgesia, patient satisfaction, pediatric anesthesia, pediatric pain, postoperative pain, postoperative pain predictors, regional analgesia, severe pain

## Abstract

Background

Postoperative pain management in children remains a significant challenge due to developmental differences in pain perception, reporting, and communication. However, limited evidence exists regarding age-specific predictors of severe pain and patient satisfaction among preschool and school-age children, particularly when perioperative, psychological, and analgesic-related factors are integrated. Despite advances in multimodal analgesia, a substantial proportion of pediatric patients continue to experience inadequate postoperative pain relief.

Objectives

To evaluate postoperative pain trajectories, identify predictors of severe pain, and assess factors associated with satisfaction in pediatric patients undergoing elective surgery, with stratification by developmental age groups (preschool versus school-age).

Methods

This prospective multicenter observational cohort study included 325 children aged three to 12 years undergoing elective surgery under general anesthesia, with or without regional anesthesia (RA). Patients were categorized into preschool (3-6 years, n=175) and school-age (6-12 years, n=150) groups. Pain intensity was assessed at predefined time points during the first 24 postoperative hours using age-appropriate validated scales: the Face, Legs, Activity, Cry, Consolability (FLACC) scale for preschool children and the Numerical Rating Scale (NRS) for school-age children. Severe pain was defined as NRS ≥7/10. Satisfaction with pain management was recorded on a 0-10 scale. Multivariable logistic regression identified independent predictors of severe pain, while linear regression evaluated factors associated with patient satisfaction.

Results

Pain scores peaked in the early postoperative period and declined significantly over time (P<0.001). Severe pain occurred in 26.5% of patients, with no significant difference between groups (P=0.324). School-age children reported higher pain immediately postoperatively, whereas preschool children exhibited higher pain scores later during recovery. Independent predictors of severe pain included higher early postoperative pain, pain at discharge, preoperative anxiety, and the need for additional analgesia. In contrast, pre-incisional local anesthetic infiltration (LAI), RA, premedication, and preoperative education were associated with lower odds of severe pain (all P<0.05). Satisfaction scores were high (mean 8.1±1.1) and were positively associated with effective pain relief, use of regional techniques, and patient/parent involvement in care. Conversely, persistent severe pain, higher early postoperative pain, and the need for additional analgesia were associated with lower satisfaction. Regression models demonstrated strong predictive performance, with Nagelkerke R² values up to 0.884 and an adjusted R² of 0.434.

Conclusion

Postoperative pain outcomes in children are predominantly associated with modifiable perioperative factors rather than age alone. Early pain control, multimodal analgesia, RA, and patient-centered strategies, including preoperative education and shared decision-making, are consistently associated with improved pain outcomes and satisfaction. These findings support the development of risk-stratified, individualized approaches to pediatric perioperative pain management.

## Introduction

Effective control of postoperative pain in children remains a persistent challenge in perioperative care. Despite advances in multimodal analgesic strategies, a considerable proportion of pediatric patients continue to experience moderate-to-severe postoperative pain after surgery, with previous studies reporting inadequate pain control in approximately 40%-70% of pediatric patients during the first 24 postoperative hours [1,2]. Inadequate pain control early in life has also been associated with long-term consequences, including altered pain sensitivity and an increased risk of chronic pain [3].

Pain assessment and management in children are inherently complex and are influenced by developmental, communication, and behavioral factors. Younger children, particularly those in the preschool age group, often lack the cognitive and verbal skills required to describe pain accurately [4]. In contrast, older school-age children are generally capable of self-reporting pain. Validated pediatric pain assessment tools, including the Face, Legs, Activity, Cry, Consolability (FLACC) scale for younger children and the Numerical Rating Scale (NRS 0-10) for older cooperative children, are commonly used [5,6]. However, children's expressions of pain may still be influenced by preoperative anxiety, prior experiences, and expectations. These developmental differences complicate both the recognition and treatment of postoperative pain across age groups [7].

Previous studies have identified a range of factors associated with increased postoperative pain in pediatric populations, including preoperative anxiety, type and duration of surgery, and variability in analgesic strategies [8]. Conversely, perioperative interventions such as regional anesthesia (RA), local anesthetic infiltration (LAI), and preoperative education have been associated with improved pain control [9,10].

Patient satisfaction is increasingly recognized as a patient-centered outcome reflecting the overall quality of perioperative care. It may be operationally defined as the individual's overall appraisal of postoperative pain management, incorporating perceived pain relief, communication, expectation alignment, and participation in decision-making. Satisfaction is influenced not only by pain relief but also by communication, expectation management, and involvement in decision-making [11,12]. However, evidence examining the relationship between pain outcomes and satisfaction in pediatric populations remains limited. Moreover, most studies evaluate isolated outcomes rather than adopting a comprehensive, patient-centered framework.

Aim of the study

This study aimed to provide a comprehensive evaluation of postoperative pain trajectories, identify predictors of severe pain, and determine factors associated with satisfaction among pediatric patients undergoing elective surgery. By stratifying patients into preschool and school-age groups, the study further explores the influence of developmental stage on pain perception, communication ability, coping, and self-report reliability, which may affect postoperative pain assessment and satisfaction.

## Materials and methods

Study design

This was a prospective, multicenter observational cohort study conducted at three tertiary care hospitals participating in a quality improvement project on postoperative pain management. The study was conducted in Saudi Arabia (King Abdulaziz and King Khalid University Hospitals, King Saud University), Egypt (Kasr Al-Ainy Hospital, Cairo University), and the United States (NEMA Research Inc.).

Data were collected using a standardized protocol to evaluate postoperative pain outcomes during the first 24 hours following elective pediatric surgery. The study adhered to the Strengthening the Reporting of Observational Studies in Epidemiology (STROBE) guidelines [13].

Ethical considerations

This study was conducted as part of a quality improvement project aimed at improving postoperative pain outcomes. Ethical approval was obtained from the Institutional Review Board of the College of Medicine, King Saud University (Ref. No. 18/0443/IRB). The study was registered at a clinical trial website (NCT05624502; registration date: January 1, 2019). Written informed consent was obtained from parents or legal guardians before enrollment in the study.

Participants

A total of 325 pediatric patients aged three to 12 years undergoing elective surgery under general anesthesia, with or without RA, were included through consecutive sampling of eligible children during the study period. Data were collected between October 2020 and December 2025.

Inclusion criteria were male and female patients with American Society of Anesthesiologists (ASA) physical status I-II, an expected postoperative stay of >6 hours, and the ability to complete the pain assessment method. Exclusion criteria included chronic opioid use, known allergy to standard analgesics, developmental or neurological disorders affecting pain assessment, major systemic diseases, refusal to participate, and incomplete patient data or incomplete follow-up during the first 24 hours.

Group assignment

The study included 325 children aged three to 12 years, who were categorized into a preschool group (3-6 years, early childhood) and a school-age group (>6-12 years, middle childhood). This classification was selected because of clinically relevant differences in pain perception, communication, and coping strategies, in accordance with the pediatric pain literature [7,11,12].

Outcome measures

The primary outcomes were predictors of severe postoperative pain and patient satisfaction with overall postoperative pain management. Severe pain was defined as NRS ≥7/10. Secondary outcomes included postoperative pain trajectories over 24 hours, worst pain scores, duration of severe pain, need for additional analgesia, and overall pain relief.

Perioperative management

Perioperative care followed institutional multimodal analgesia protocols. All patients received general anesthesia with inhalational and intravenous agents, supplemented by opioids and non-opioid analgesics. The relevant weight-based doses used in our protocol included fentanyl 1-2 mcg/kg IV and ketamine 0.5-1 mg/kg IV when used to provide intraoperative analgesia.

RA techniques included ultrasound-guided transversus abdominis plane (TAP) block, peripheral nerve block (PNB), or neuraxial block (caudal epidural), which were used when appropriate. Additional analgesic strategies included pre-incisional LAI, fentanyl, paracetamol, non-steroidal anti-inflammatory drugs (NSAIDs), and ketamine.

Postoperative analgesia included scheduled paracetamol with or without NSAIDs, with opioid rescue therapy (e.g., fentanyl or morphine) as required. Non-pharmacological strategies (e.g., distraction, parental presence, and VR) were recorded when applied.

Data collection

Preoperative data, including baseline demographic and clinical characteristics, were collected during the routine preoperative assessment: age (years), age group, gender, BMI (kg/m²), ASA physical status, and type of procedure (e.g., general surgery, orthopedic, ENT, or dental). Preoperative predictors included preoperative pain during the preceding three months and preoperative anxiety. Preoperative anxiety was assessed clinically during the preoperative anesthesia assessment as a preoperative clinical variable using a 0-10 scale, where 0 indicated no anxiety, while 10 indicated extreme anxiety, rather than using a formal anxiety scale. The anesthesia team provided education on the anesthetic plan, pain assessment tools, and possible modalities for postoperative pain management, and recorded parents’ or older children’s participation in shared decision-making regarding analgesic options.

Intraoperative variables included pre-incisional LAI, anesthetic technique (general anesthesia alone vs. combined with RA), intraoperative multimodal analgesia (including intraoperative fentanyl dose, paracetamol, NSAIDs, and ketamine use), and duration of surgery (minutes).

Postoperative data included pain intensity, which was assessed using age-appropriate tools based on developmental ability. Preschool children were assessed using the FLACC observational scale, whereas school-age children used the self-reported NRS [5,6]. For analysis, both scales were expressed on a common 0-10 framework to facilitate comparison across age groups; this represented score harmonization rather than a validated psychometric conversion. Pain scores were recorded in the post-anesthesia care unit (PACU), at admission to the day surgery unit (DSU) or ward, and at three, six, 12, and 24 hours postoperatively, as well as at discharge. Additional pain outcomes included pain on activity, pain during sleep, worst pain, and least pain during the first 24 hours, duration of time spent at worst pain (minutes), and whether the child experienced severe pain.

Requests for additional analgesia (“need more analgesia”) and rescue analgesic doses were recorded. Global experience of overall pain relief was recorded on a 0-100 scale (0=no relief, 100=complete relief). Satisfaction with overall pain management was assessed using a numeric scale from 0 (extremely dissatisfied) to 10 (excellent). For school-age children, the scales were completed by the child when feasible, whereas for preschool children, they were reported using a child-mother composite assessment to enhance the quality of the evaluations.

Sample size calculation

Based on prior literature, a 1-point difference on the NRS was considered clinically meaningful. Assuming an SD of 2 points, α = 0.05, and 80% power, a minimum of 64 patients per group was required to detect this difference between the preschool and school-age groups.

To improve statistical robustness and enable multivariable modeling, a larger cohort (n=325) was included. This approach enhanced precision and supported reliable identification of independent predictors.

Statistical analysis

Data were analyzed using SPSS version 27 (IBM Corp., Armonk, NY, USA). Continuous variables were summarized as mean ± SD. Categorical variables were summarized as counts and percentages. Comparisons between preschool and school-age children were performed using the independent-samples t-test for normally distributed continuous variables and the Mann-Whitney U test for non-normally distributed continuous variables. Categorical variables were compared using the Chi-Square test.

Correlation analyses were conducted to explore associations between perioperative predictors and pain outcomes. Pearson correlation coefficients were used for pairs of continuous, approximately normally distributed variables, whereas Spearman rank correlation coefficients were used when at least one variable was ordinal or binary.

Logistic regression analysis was performed to identify independent predictors of severe pain. Results are presented as odds ratios (ORs) with 95% confidence intervals (CIs). Model performance was evaluated using Nagelkerke pseudo-R² and the area under the receiver operating characteristic (ROC) curve. Linear regression was also conducted to identify predictors of satisfaction with pain management (β coefficients).

Model performance was assessed using Nagelkerke R² and AUC (ROC analysis). A two-sided P<0.05 was considered statistically significant.

## Results

The study enrolled 325 pediatric patients aged three to 12 years undergoing elective surgical procedures under general anesthesia. Patients were classified into two groups as follows: 175 (53.8%) preschool children (3-6 years) and 150 (46.2%) school-age children (>6-12 years). Patient characteristics, perioperative data, and pain outcomes are summarized in Tables 1, 2, 3.

**Table 1:** Baseline characteristics of the study participants ^*^Data expressed as a number (%) and compared by the Chi-Square test. P<0.05 is significant. Data expressed as mean (+SD) and comparison between the groups by an independent t-test. BMI: body mass index; ASA: American Society of Anesthesiologists; M/F: male/female; ENT: ear, nose, and throat; Ortho: orthopedic; GS: general surgery

Variables	All patients	Preschool	School age	P
Number of participants	325	175 (53.8%)	150 (46.2%)	-
Age/year (mean+SD)	6.82+2.6	4.83+0.9	9.15+1.9	<0.001
Gender N(%)^*^ M/F	183 (56.3%)/142 (43.7%)	103 (58.9%)/72 (41.1%)	80 (53.3%)/70 (46.7%)	0.370
BMI (kg/m²)	20.57+4.1	20.02+3.9	21.2+4.1	0.009
ASA physical status N(%)^*^ I/II	275 (84.6%)/50 (15.4%)	146 (83.4%)/29 (16.6%)	129 (86%)/21 (14%)	0.541
Preoperative chronic pain^*^	31 (9.5%)	19 (10.8%)	12 (8%)	0.451
Preoperative anxiety^*^	80 (24.6%)	49 (28%)	31 (20.7%)	0.040
Preoperative education^*^	256 (78.8%)	134 (76.6%)	122 (81.3%)	0.341
Patient/parent participation in decision-making^*^	225 (69.2%)	110 (62.9%)	115 (76.7%)	0.008
Premedication	141 (43.4%)	84 (48%)	57 (38%)	0.044
Duration of surgery (min)	99.57+25.9	101.5+24.6	97.3+27.3	0.151
Surgical procedures				
ENT	124 (38.2%)	60 (34.3%)	64 (42.7%)	0.514
Ortho	76 (23.4%)	46 (26.3%)	30 (20%)	0.030
GS	75 (23.1%)	34 (19.4%)	41 (27.3%)	0.045
Dental	50 (15.4%)	35 (20%)	15 (10%)	0.015

**Table 2:** Perioperative data of the study groups ^*^Data expressed as a number (%) and compared by the Chi-Square test. P<0.05 is significant. Data expressed as mean+SD and comparison between the groups by an independent t-test. GA: general anesthesia; RA: regional anesthesia; LAI: local anesthesia infiltration; PNB: peripheral nerve block; TAP: transversus abdominis plane; VR: virtual reality; NSAIDs: non-steroidal anti-inflammatory drugs

Variables	All patients	Preschool	School age	P
Type of anesthesia N(%)^*^ GA/GA+RA	255 (78.5%)/70 (21.5%)	133 (76%)/42 (24%)	122 (81.3%)/28 (18.7%)	0.014
Pre-incisional LAI^*^	173 (53.2%)	94 (53.7%)	79 (52.7%)	0.469
RA techniques				
PNB^*^	31 (9.5%)	23 (13.14%)	8 (5.33%)	0.040
TAP block^*^	25 (7.7%)	10 (3.07%)	15 (5.3%)	0.041
Caudal epidural^*^	14 (4.3%)	9 (5.7%)	5 (3.3%)	0.460
Intraoperative fentanyl (µg)	47.6+16.9	44.17+16.4	51.6+15.7	0.017
Intraoperative NSAIDs N(%)	60 (18.5%)	29 (16.6%)	31 (20.7%)	0.800
Intraoperative ketamine N(%)	98 (30.2%)	50 (28.6%)	48 (32%)	0.794
Intraoperative paracetamol (mg)	428.0+153.5	363.4+8.2	503.3+13.3	<0.010
Postoperative morphine N(%)	83 (25.5%)	36 (20.6%)	47 (31.3%)	0.030
Non-pharmacological tools: N(%)				
No intervention	202 (62.1%)	92 (52.6%)	110 (73.3%)	0.010
Mobile phone	51 (15.6%)	30 (17.14%)	21 (14.0%)	
Video games	47 (14.5%)	31 (17.6%)	16 (10.7%)	
Presence of parent(s)	19 (5.8%)	17 (9.7%)	2 (1.33%)	
VR	6 (1.8%)	4 (2.3%)	2 (1.33%)	

**Table 3:** Pain outcomes and patient satisfaction among the study participants ^*^Data are expressed as numbers (%) and compared by the Chi-Square test. Data expressed as mean+SD and comparison between the groups by an independent t-test. P<0.05 is significant. PACU: post-anesthesia care unit

Variables	All Patients	Preschool	School age	P
Pain in PACU	2.84+1.9	2.64+1.8	3.1+2.1	0.047
Pain on admission to the ward	2.22+1.5	2.54+1.6	1.85+1.1	<0.01
After 1 hour	2.15+1.06	2.21+1.1	1.96+0.9	0.066
After 3 hours	1.85+1.3	1.93+1.4	1.75+1.1	0.197
After 6 hours	1.57+1.2	1.7+1.2	1.41+1.1	0.021
After 12 hours	1.61+1.2	1.78+1.3	1.41+1.08	0.006
After 24 hours	1.33+0.8	1.48+0.9	1.16+0.7	<0.01
Pain at discharge	1.35+0.7	1.48+0.7	1.21+0.7	<0.01
Least pain score (in 24 h)	0.92+0.72	1.02+0.8	0.8+0.6	0.007
Worst pain score (in 24 h)	4.18+1.87	4.14+1.9	4.26+1.8	0.757
Severe pain^*^	86 (26.5%)	44 (25.1%)	42 (28%)	0.324
Duration of severe pain	25.83+14.2	26.1+14.6	25.5+13.7	0.726
Pain with activity	3.5+1.6	3.46+1.6	3.54+1.6	0.805
Pain during sleep^*^	69 (21.2%)	42 (24.0%)	27 (18.0%)	0.221
Need more analgesia^*^	61 (18.76%)	29 (16.6%)	32 (21.3%)	0.170
Overall pain relief (%)	80.6+9.6	80+10.3	81.2+8.7	0.281
Satisfaction	8.1+1.1	8.02+1	8.27+1	0.044

Patient characteristics and perioperative data

The mean age of all patients was 6.82±2.6 years, with significant differences between the two groups (preschool: 4.8±0.9 years; school-age: 9.15±1.9 years; P<0.001). BMI was slightly higher in school-age children (P=0.009), while sex distribution, ASA status (I or II), preoperative chronic pain, and preoperative patient education were comparable between the groups (P>0.05). Preoperative anxiety was reported in 24.6% of patients, with slightly higher rates in preschool children (P=0.04). The study included four types of surgical procedures: ENT (38.2%), orthopedic (23.4%), general surgery (23.1%), and dental (15.4%), with significant group differences for orthopedic, general surgery, and dental cases (Table 1).

Perioperative analgesia

Intraoperative analgesia was provided according to the anesthetic technique; general anesthesia alone was used in 78.5% of patients, with regional augmentation (PNB, TAP block, or caudal epidural) in 21.5% (P<0.05 between groups). Pre-incisional LAI was used in 53.2% of cases, without differences between groups (P=0.469). Intraoperative fentanyl, paracetamol, and NSAIDs were widely used within weight-adjusted pediatric dose ranges, whereas intraoperative ketamine was used in 30.2% of patients.

Postoperatively, nearly all children received scheduled paracetamol, and many received NSAIDs when not contraindicated. Rescue opioids (morphine or equivalent doses of fentanyl) were used in 25.5% of patients, with a higher incidence in the older school-age group (P=0.03). Non-pharmacological interventions were applied in 37.9% of all patients (e.g., mobile phone distraction in 15.6%, video games in 14.5%, parental presence in 5.8%, and virtual reality in 1.8%) (Table 2).

Postoperative pain trajectories

Postoperative pain scores followed a dynamic trajectory and decreased significantly over time, with peak intensity occurring in the immediate postoperative period. Pain peaked in the PACU (mean 2.84±1.9) and declined thereafter, with significant changes over time across the first 24 hours (P<0.001). School-age children reported higher immediate postoperative pain (in the PACU). No significant differences between the groups were observed at one and three hours postoperatively (P>0.05). In contrast, the preschool group demonstrated higher pain scores later during recovery, including at DSU/ward arrival and at six, 12, and 24 hours postoperatively, as well as at discharge (all P<0.05), potentially reflecting developmental differences in pain expression and reporting.

Worst pain during the first 24 hours was moderate (4.18±1.87), without group differences (P=0.757). Severe pain occurred in 26.5% of patients (86/325), with no group difference (P=0.324). The mean duration of severe pain was 25.83±14.2 minutes, with no significant differences between groups (P=0.726). Activity-related pain was 3.5±1.6. Pain that awakened patients during sleep affected 21.2% of patients, with a mean pain score of 3.5±1.6; no group differences were observed (P=0.221). The need for additional analgesia was reported in 18.76% of patients, with no difference between the two groups (P=0.170).

**Figure 1:**
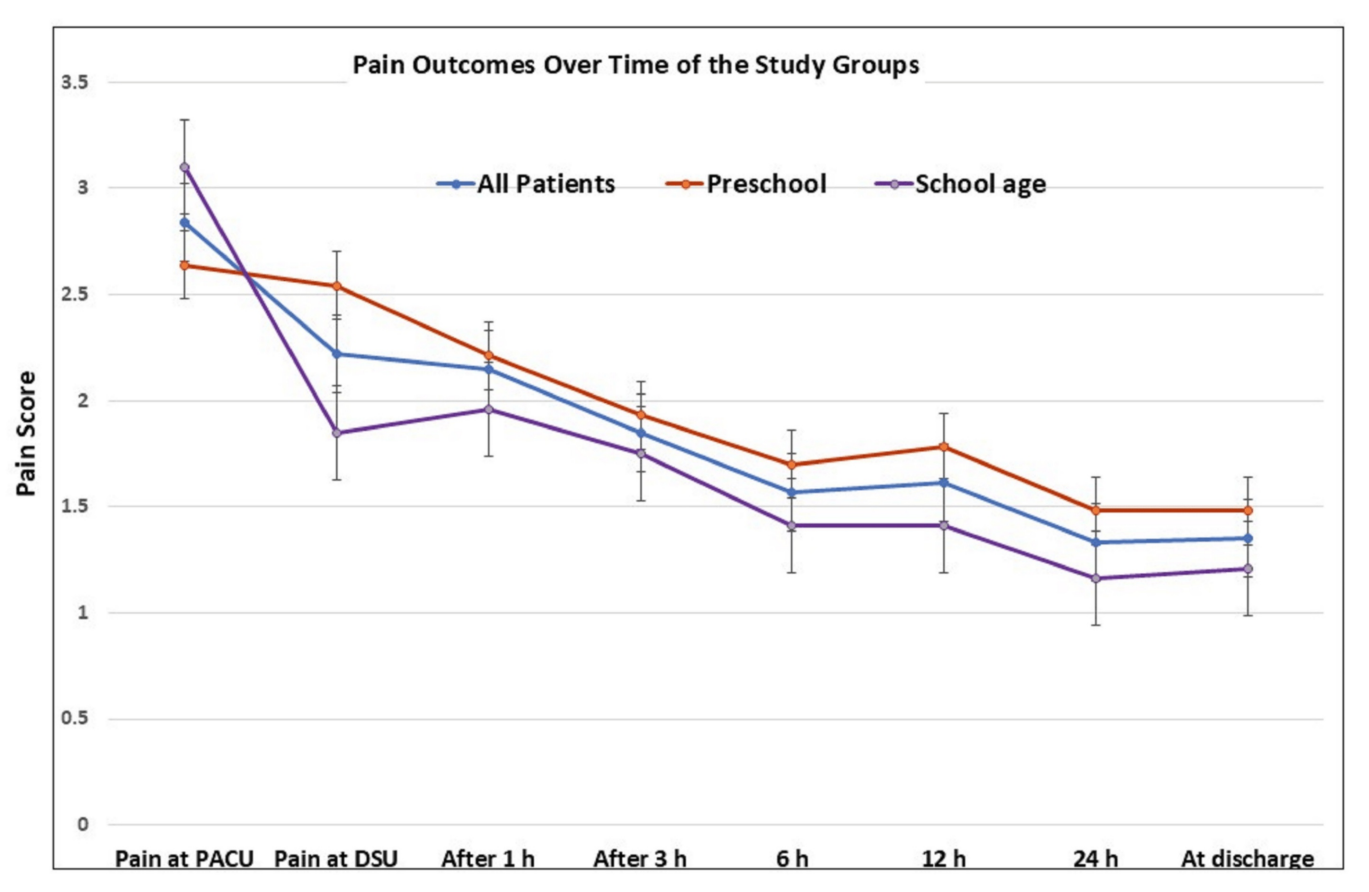
Pain outcomes over time of the study groups Data expressed as mean+SD PACU: post-anesthesia care unit, DSU: day surgery unit

Overall pain relief was 80.6±9.6, and the patient satisfaction score was 8.1±1.1, with significantly higher satisfaction in school-age children (P=0.019). The least pain during the first 24 hours was 0.92±0.72 in all patients, with a significantly lower pain score in the school-age group (P=0.007) (Table 3 and Figure 1).

Correlation analysis

**Figure 2:**
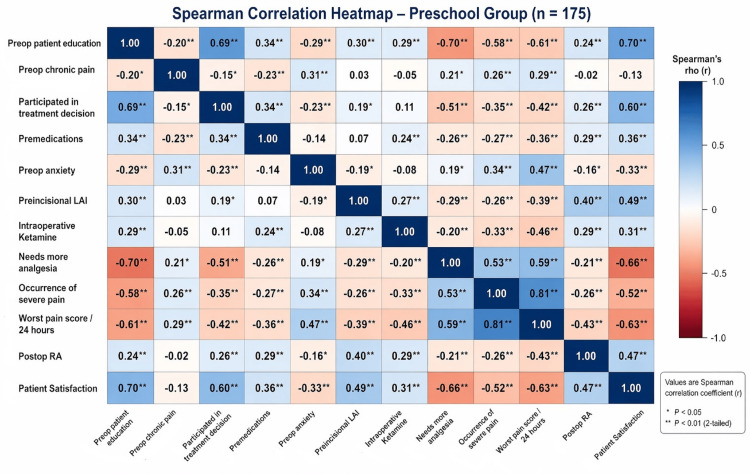
Correlation heatmap for predictors of severe pain and patient satisfaction in the preschool group LAI: local anesthetic infiltration; Preop: preoperative; Postop: postoperative; RA: regional anesthesia

**Figure 3:**
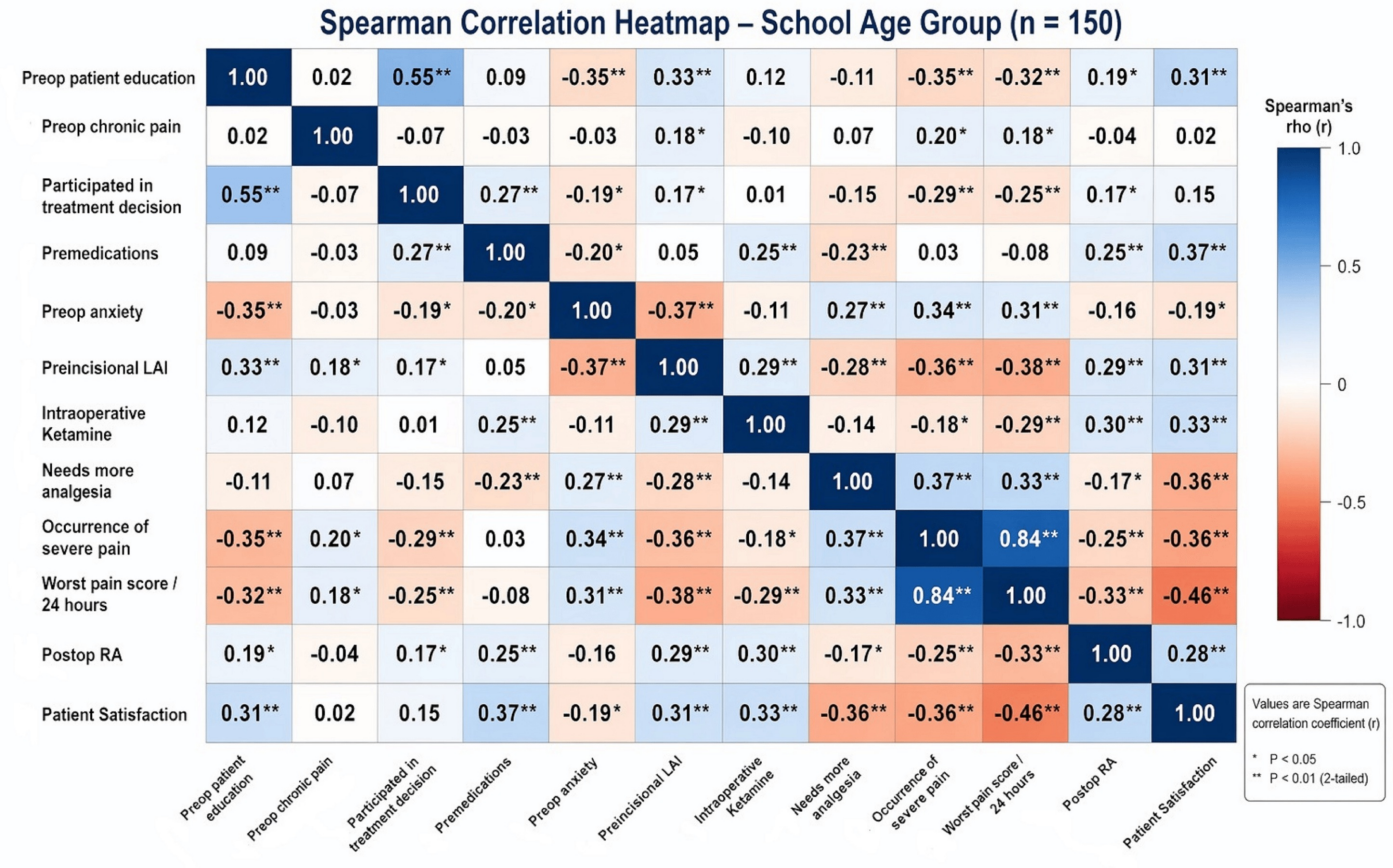
Correlation heatmap for predictors of severe pain and patient satisfaction in the school group LAI: local anesthetic infiltration; Preop: preoperative; Postop: postoperative; RA: regional anesthesia

Correlation analysis showed consistent patterns between pain outcomes and perioperative variables across groups. Worst pain showed a strong positive correlation with severe pain (r=0.81 to 0.84, P<0.01), need for analgesia (r=0.59 to 0.33, P<0.01), and the need for more analgesia with the occurrence of severe pain (r=0.53 to 0.37, P<0.05), while worst pain was negatively correlated with patient satisfaction (r=-0.63 to -0.46, P<0.05) in the preschool and school-age groups, respectively (Figures 2, 3).

Protective factors such as pre-incisional LAI were negatively correlated with severe pain (r=-0.39 to -0.38, P<0.05) and worst pain (r=-0.39 to -0.38, P<0.05), and positively correlated with satisfaction (r=0.49 to 0.31, P<0.05). Preoperative anxiety showed positive correlations with worst pain and severe pain (r=0.47 to 0.31, P<0.05) in the preschool and school-age groups, respectively (Figures 2, 3).

Analgesic interventions demonstrated protective associations. Postoperative RA was negatively correlated with worst pain (r=-0.43 to -0.33, P<0.05) and positively correlated with satisfaction (r=0.47 to 0.28, P<0.05). Similarly, preoperative education and patient/parent participation in decision-making were associated with improved satisfaction and overall pain relief (Figures 2, 3).

Regression analysis

Logistic Regression Analysis for Predictors of Severe Pain

Multivariable logistic regression identified several independent predictors of severe postoperative pain. In the preschool group, premedication and pre-incisional LAI were significantly associated with reduced odds of severe postoperative pain. Whereas uncontrolled worst pain, need for additional analgesia, preoperative anxiety, and early postoperative pain in the PACU were associated with increased odds of severe pain.

In the school-age group, worst postoperative pain, need for additional analgesia, and preoperative anxiety were significant predictors of severe pain, while postoperative RA, intraoperative ketamine, and pre-incisional LAI demonstrated significant protective effects.

**Figure 4:**
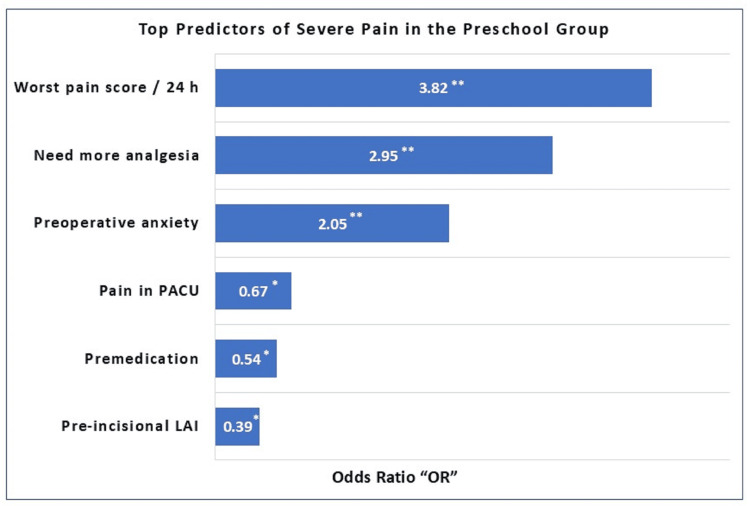
Logistic regression analysis of the top predictors of severe postoperative pain in the preschool group ^*^Statistical significance at P<0.05. ^**^Statistical significance at P<0.01. LAI: local anesthesia infiltration; PACU: post-anesthesia care unit

**Figure 5:**
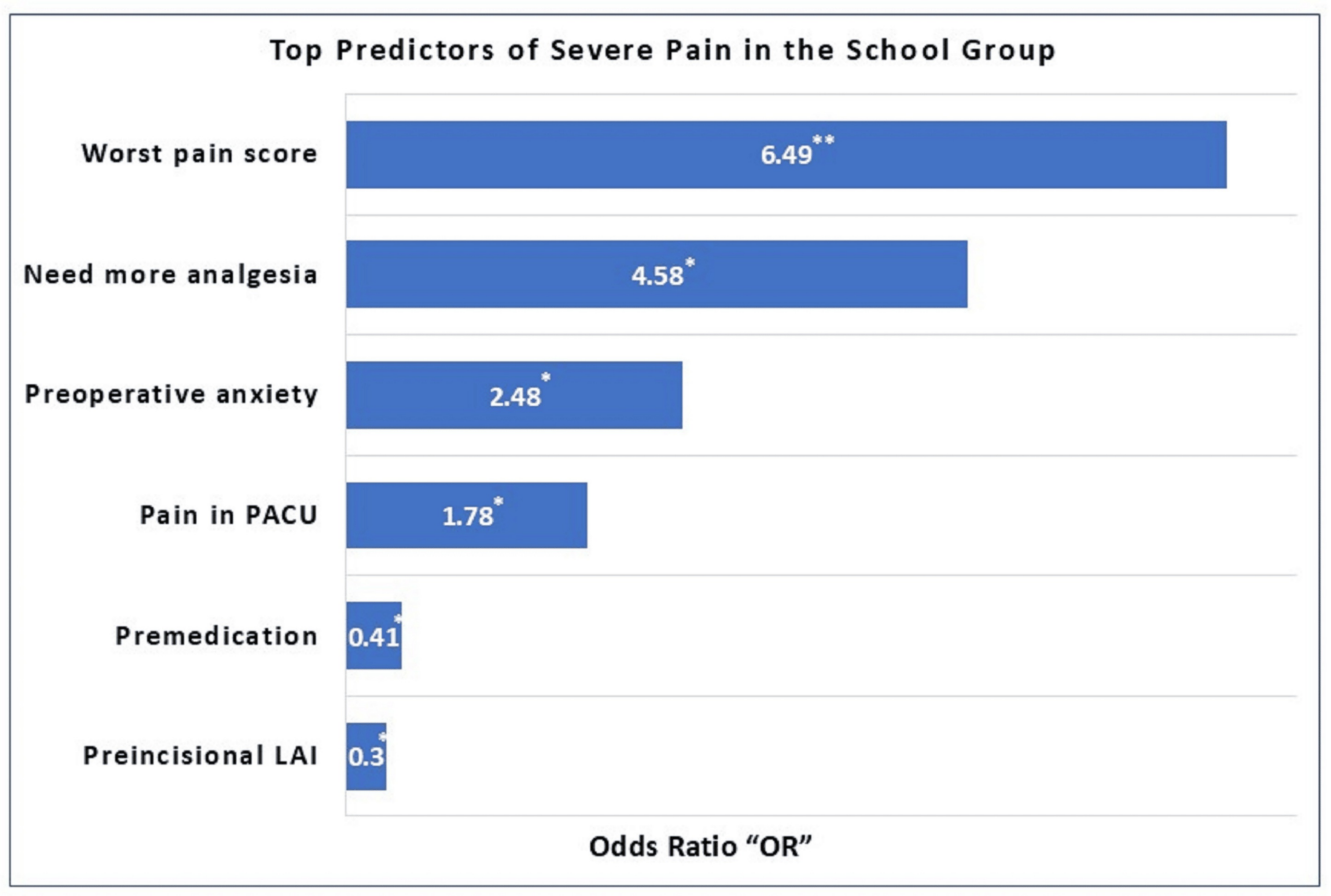
Logistic regression analysis of the top predictors of severe postoperative pain in the school group ^*^Statistical significance at P<0.05. ^**^Statistical significance at P<0.01. LAI: local anesthesia infiltration; PACU: post-anesthesia care unit

Model performance demonstrated good predictive ability, with high explanatory power. In the preschool group, the model demonstrated good discriminative ability, with a Nagelkerke R² of 0.68 and an AUC of 0.89 (95% CI 0.83-0.95), indicating excellent discrimination ability. Similarly, in the school-age group, the model showed good explanatory power (Nagelkerke R²=0.61) and excellent discrimination (AUC=0.92; 95% CI 0.87-0.97), indicating robust predictive accuracy across developmental stages. The Hosmer-Lemeshow test was non-significant in both groups (P>0.05), indicating excellent model calibration, meaning that the models’ predictions aligned well with the actual outcomes (Table 4, Figures 4, 5).

**Table 4:** Multivariable logistic regression analysis of independent predictors of severe postoperative pain P-value <0.05 is considered significant. Model performance Preschool group: Nagelkerke R²=0.68, Hosmer-Lemeshow P=0.72 (>0.05), and AUC (95% CI)=0.89 (0.83-0.95). School-age group: Nagelkerke R²=0.61, Hosmer-Lemeshow P=0.81 (>0.05), and AUC (95% CI)=0.92 (0.87-0.97). OR: odds ratio; CI: confidence interval; AUC: area under the receiver operating characteristic curve; LAI: local anesthetic infiltration; RA: regional anesthesia; PACU: post-anesthesia care unit

(A) Preschool group					
Predictor	β	OR (Expβ)	95% CI	P-value	
Worst pain score/24 h	1.87	6.49	2.10-20.10	0.001	
Patient needs more analgesia	1.52	4.58	1.60-13.10	0.004	
Preoperative anxiety	0.91	2.48	1.30-4.75	0.006	
Pain in PACU	0.58	1.78	0.93-3.41	0.036	
Premedication	-0.88	0.41	0.18-0.93	0.033	
Pre-incisional LAI	-1.21	0.30	0.12-0.74	0.009	
(B) School-age group					
Predictor	β	OR (Expβ)	95% CI	P-value	
Worst pain score/24 h	1.34	3.82	1.90-7.65	<0.001	
Patient needs more analgesia	1.08	2.95	1.50-5.80	0.002	
Preoperative anxiety	0.72	2.05	1.15-3.65	0.015	
Postoperative RA	-1.85	0.67	0.17-1.37	0.039	
Intraoperative ketamine	-0.61	0.54	0.30-0.98	0.041	
Pre-incisional LAI	-0.95	0.39	0.20-0.76	0.048	

Before finalizing the regression models, predictor relationships were examined to ensure that variable overlap was not affecting model performance. Variance inflation factor analysis showed no concerning collinearity, with all retained predictors remaining within acceptable limits. While some perioperative variables were clinically related, each contributed independently within the multivariable model and was retained based on both clinical importance and statistical contribution. The relatively high predictive performance likely reflects the inclusion of early postoperative pain measures and perioperative analgesic variables, which are closely related to later pain severity and therefore expected to explain a substantial proportion of the observed variation.

Linear Regression Analysis for Predictors of Patient Satisfaction

Multivariate linear regression analyses were performed to identify key predictors of patient satisfaction in both age groups.

In the preschool group, higher satisfaction was significantly associated with preoperative education, pre-incisional LAI, postoperative RA, and non-pharmacological tools (e.g., mobile phones, video games, and VR). Conversely, lower satisfaction was associated with uncontrolled postoperative pain and the need for additional analgesia. Higher pain scores on admission to the ward/DSU, longer duration of severe pain, and preoperative anxiety were associated with decreased satisfaction, but the results were non-significant.

In the school-age group, higher satisfaction was significantly associated with preoperative patient education, patient/parent participation in decision-making, pre-incisional LAI, and intraoperative ketamine. In contrast, lower satisfaction was significantly associated with longer duration of severe pain, need for additional postoperative analgesia, and uncontrolled worst pain. Higher pain scores in the PACU, duration of surgery, pain at discharge, and pain with activity showed trends toward association but did not reach statistical significance.

In the preschool group, the top predictors of higher satisfaction were preoperative patient/parent education (B=0.48, P<0.001) and pre-incisional LAI (B=0.36, P<0.001). The strongest negative predictors were uncontrolled worst pain (B=-0.52, P=0.001) and the need for additional analgesia (B=-0.41, P=0.002).

In the school-age group, the strongest negative predictor was the worst postoperative pain score (B=-0.46, P<0.011), followed by the need for additional analgesia (B=-0.34, P=0.014) and duration of severe pain (B=-0.311, P=0.013). Preoperative patient education (B=0.31, P=0.026), pre-incisional LAI (B=0.29, P<0.001), and intraoperative ketamine (B=0.27, P=0.023) were positive predictors of patient satisfaction.

**Figure 6:**
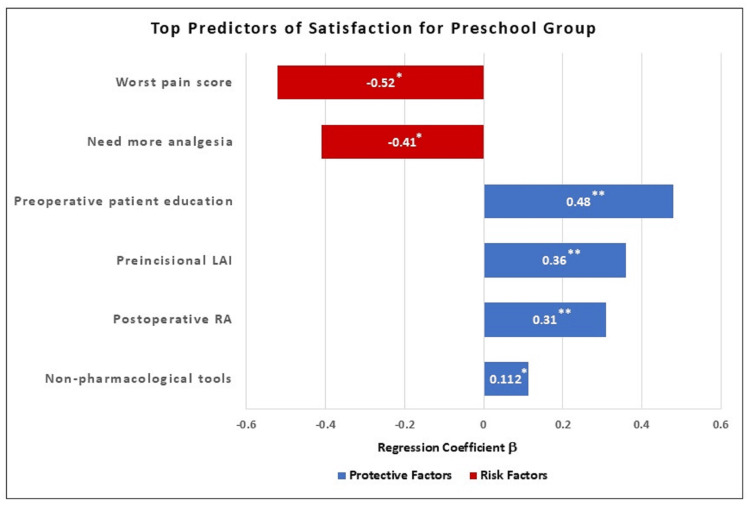
Linear regression analysis of the top predictors of patient satisfaction for the preschool group ^*^Statistical significance at P<0.05. ^**^Statistical significance at P<0.01. LAI: local anesthesia infiltration; RA: regional anesthesia

**Figure 7:**
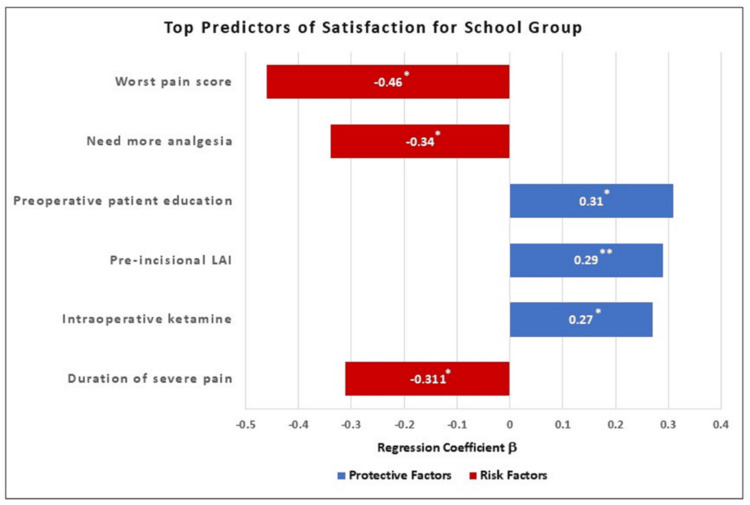
Linear regression analysis of the top predictors of patient satisfaction for the school-age group ^*^Statistical significance at P<0.05. ^**^Statistical significance at P<0.01. LAI: local anesthesia infiltration.

The models demonstrated good overall performance. In the preschool group, the model showed good explanatory capacity, accounting for 64% of the variance in patient satisfaction (R²=0.64; adjusted R²=0.62). Similarly, in the school-age group, the model explained a moderate proportion of the variability in satisfaction (R²=0.57; adjusted R²=0.55), indicating that satisfaction is influenced by a combination of pain-related outcomes and perioperative care factors (Table 5, Figures 6, 7).

**Table 5:** Multivariable linear regression analysis of predictors of patient satisfaction P-value <0.05 is considered significant. Model performance Preschool group: R²=0.64; Adjusted R²=0.62 School-age group: R²=0.57; Adjusted R²=0.55 β: standardized regression coefficient; CI: confidence interval; SE: standard error; LAI: local anesthesia infiltration; RA: regional anesthesia

(A) Preschool group				
Predictor	β standardized	95% CI	SE	P-value
Worst pain score/24 h	-0.52	-0.66--0.38	0.054	0.001
Patient needs more analgesia	-0.41	-0.55--0.26	0.169	0.041
Preoperative patient education	0.48	0.34 - 0.60	0.192	<0.001
Pre-incisional LAI	0.36	0.21-0.50	0.107	<0.001
Postoperative RA	0.31	0.17-0.45	0.129	<0.001
Non-pharmacological tools	0.112	0.072-0.605	0.048	0.035
(B) School-age group				
Predictor	β standardized	95% CI	SE	P-value
Worst pain score/24 h	-0.46	-0.58--0.33	0.049	0.011
Patient needs more analgesia	-0.34	-0.48--0.20	0.191	0.014
Preoperative patient education	0.31	0.18-0.45	0.217	0.026
Pre-incisional LAI	0.29	0.15-0.43	0.155	<0.001
Intraoperative ketamine	0.27	0.12-0.41	0.147	0.023
Duration of severe pain	-0.311	-0.033--0.014	0.05	0.013

## Discussion

This multicenter prospective observational study provides a comprehensive evaluation of postoperative pain trajectories, predictors of severe pain, and determinants of patient satisfaction in pediatric surgical patients, highlighting modifiable perioperative factors across two key developmental stages (preschool and school-age).

This study offers a broader clinical perspective than many previous pediatric postoperative pain reports by evaluating not only pain intensity over time, but also predictors of severe pain and determinants of satisfaction within the same perioperative model. A key gap in the literature is that most previous pediatric studies have focused on single analgesic techniques, selected procedures, or single postoperative time points, rather than integrating pain trajectories, predictors of severe pain, and satisfaction outcomes within one developmental framework [8,10,14]. The present analysis addresses this gap by examining how multiple perioperative variables interact across developmental stages in routine clinical practice, and the consistent identification of modifiable factors such as pre-incisional LAI, RA, premedication, and preoperative education strengthens the clinical relevance of the findings and supports a more individualized approach to pediatric perioperative pain management.

Several important findings emerged from this study, supported by previous studies. First, despite advances in multimodal analgesia and evolving perioperative pain management strategies, postoperative pain remains inadequately controlled in a proportion of pediatric patients, consistent with previous reports [15,16]. Second, pain outcomes were primarily driven by modifiable perioperative variables rather than fixed patient characteristics, aligning with findings from adult surgical populations, where early pain control and perioperative management strategies are key determinants of outcomes [4,17,18]. Third, although this study showed that the incidence of severe postoperative pain (26.5%) was lower than that reported in many previous cohorts, a clinically meaningful proportion of children continued to experience suboptimal pain control, highlighting persistent gaps in care [2,19,20].

Developmental considerations and the effect of age group

Pain trajectories demonstrated clear developmental differences. School-age children reported higher pain scores during mobilization and in the immediate postoperative period, likely reflecting a greater ability to self-report pain and possibly heightened anticipatory anxiety [6,21]. In contrast, preschool children exhibited higher pain scores later during recovery, which may reflect delayed recognition, limited communication abilities, or behavioral expression of discomfort. These findings emphasize the need for age-tailored pain assessment strategies [5,21,22].

Importantly, the developmental stage appeared to influence pain expression and reporting rather than intrinsic pain severity, consistent with prior literature [16,23]. After adjustment for confounders, age group was not an independent predictor of severe pain, suggesting that clinical and perioperative factors outweigh age alone in determining outcomes [24]. While modest associations between the school-age group and selected outcomes were observed, these findings reinforce that age should be considered within a broader biopsychosocial framework rather than as a standalone determinant [25].

Predictors of severe postoperative pain

Overall, both logistic and linear regression models demonstrated good performance, with strong explanatory power and appropriate model fit across preschool and school-age groups.

The incidence of severe pain in this cohort was 26.5%, which is numerically lower than the rates reported in earlier pediatric cohorts, where moderate-to-severe pain frequently exceeded 30%-50% [26,27]. Although the rate in our study appears lower, this comparison should be interpreted cautiously, as the difference between the two groups in our results was not statistically significant. This finding may reflect ongoing improvements in multimodal analgesia and greater use of regional techniques. However, nearly one in four children still experienced severe pain, indicating that current strategies remain insufficient for a substantial subset of patients.

The predictive performance of the multivariable models was further supported by ROC curve analysis, which demonstrated excellent discrimination in both pediatric subgroups. The preschool model achieved an AUC of 0.89, while the school-age model achieved an AUC of 0.92, indicating a strong to outstanding ability to distinguish between patients with and without severe postoperative pain. These findings suggest that the identified predictors are not only statistically significant but also clinically useful for stratifying risk at the individual patient level.

Severe pain appeared to be more frequently reported in school-age children, likely due to improved self-reporting, whereas in preschool children, it may be under-recognized or expressed mainly through behavior [28]. Pain perception in children is also influenced by maturational changes in nociceptive processing, stress responsiveness, and descending pain modulation, which may partly explain age-related differences in pain expression and reporting. Children with severe pain were more likely to require rescue analgesia, and although the time spent in severe pain was relatively short, it was clinically meaningful and associated with greater pain burden and lower satisfaction. Furthermore, pain during activity and sleep exceeded resting pain scores, emphasizing the importance of assessing functional pain outcomes in pediatric practice [29].

Role of modifiable risk factors

Early postoperative pain (particularly in the PACU), pain at discharge, and the need for additional analgesia emerged as key predictors of severe pain. These findings are consistent with prior studies demonstrating that early pain intensity is a strong determinant of subsequent pain trajectory [30,31].

These results support a trajectory-based model of postoperative pain, in which inadequate early control leads to escalation over time. Early pain may therefore serve as a practical clinical marker for identifying high-risk patients requiring intensified analgesic strategies [16,23,32].

Preoperative anxiety was independently associated with worse pain outcomes, consistent with evidence that psychological factors amplify pain perception and increase analgesic requirements [6,33]. This highlights the importance of integrating psychological assessment and targeted interventions into perioperative care pathways [34].

RA and LAI demonstrated consistent protective effects, reducing severe pain and improving satisfaction. These findings reinforce current recommendations supporting multimodal analgesia and opioid-sparing strategies in pediatric anesthesia [10,35]. Similarly, preoperative education and shared decision-making with patients and caregivers were associated with improved outcomes, likely through reduction of anxiety, improved expectations, and enhanced engagement in care [36]. These findings support the integration of structured educational interventions as a routine component of perioperative management.

Predictors of patient satisfaction

Satisfaction was more strongly associated with effective and timely pain relief than with absolute pain intensity, consistent with prior literature [37]. Children who required additional analgesia, experienced higher early postoperative pain, or had prolonged pain episodes reported lower satisfaction.

Intraoperative ketamine emerged as a positive predictor of satisfaction in school-age children, possibly reflecting its role in reducing central sensitization and improving overall analgesic quality [38].

Positive determinants of satisfaction included premedication, preoperative education, patient and parent involvement in decision-making, and the use of RA [1,39]. Conversely, persistent severe pain and inadequate early pain control negatively affected satisfaction. These findings emphasize that patient experience is shaped not only by analgesic efficacy but also by communication, expectations, and involvement in care [37,40].

Incorporation of non-pharmacological strategies, including distraction techniques and parental involvement, may further enhance outcomes, particularly in younger children, supporting a holistic, patient-centered approach to pain management. This is supported by evidence showing that distraction-based approaches can reduce pediatric pain and anxiety during procedural and postoperative care [40-42].

Clinical implications

These findings have several important clinical implications. First, prioritizing early postoperative pain control is essential, as pain in the PACU and at discharge strongly predicts subsequent outcomes. Second, the identification of modifiable predictors supports the development of risk-stratified, individualized analgesic pathways, similar to enhanced recovery protocols in adult populations. Third, routine integration of psychological and educational interventions may address modifiable risk factors such as anxiety and unmet expectations. Finally, a multimodal approach combining pharmacological, regional, and non-pharmacological strategies remains fundamental to optimizing pediatric postoperative pain outcomes.

Strengths and limitations

This study is strengthened by its multicenter design, relatively large sample size, and comprehensive assessment of both clinical and patient-reported outcomes. However, several limitations should be acknowledged.

First, the observational design precludes causal inferences regarding the relationships between identified predictors and postoperative pain outcomes, although multivariable regression analysis was performed to adjust for potential confounders and support the clinical relevance of the findings.

Second, variability in surgical procedures may have introduced heterogeneity, although this also enhances generalizability.

Third, pain assessment in younger children remains challenging, especially when directly comparing pain intensity between younger and older children. Pain assessment was performed using age-appropriate scales: preschool children were evaluated using the FLACC scale, while school-age children who could communicate reliably used the NRS. This approach facilitates comparison, but it may introduce measurement heterogeneity, particularly between younger and older children. Nevertheless, this approach is widely accepted in pediatric postoperative pain research because both tools are validated within comparable scoring ranges and are designed to reflect clinically meaningful pain intensity despite differences in assessment method.

Finally, pain outcomes were assessed only during the first 24 postoperative hours; longer-term outcomes beyond 24 hours were not evaluated.

Future directions

Future research should focus on external validation of these findings, development of predictive models for individualized pediatric pain management, and integration of both clinical and psychological variables. Randomized trials evaluating targeted interventions, such as structured preoperative education, anxiety-reduction strategies, and optimized RA protocols, are warranted. Additionally, the integration of digital tools and real-time pain monitoring systems may further improve perioperative care delivery.

## Conclusions

Pediatric postoperative pain follows a dynamic trajectory with meaningful differences between preschool and school-age children. Although overall pain control and satisfaction were favorable, a substantial proportion of patients continued to experience severe pain. Modifiable factors, including preoperative anxiety, education, RA, and early postoperative pain control, play a central role in determining both pain outcomes and satisfaction. Targeting these factors through structured, multimodal, and patient-centered strategies offers a practical pathway to improving perioperative care.

Based on the findings, a tailored, developmentally informed approach integrating clinical, psychosocial, and educational components is recommended to optimize outcomes and enhance the patient experience. The identified predictors should be regarded as hypothesis-generating and may inform the design of future interventional studies aimed at improving pediatric postoperative pain management.
